# Non-Contact Measurement of Human Vital Signs in Dynamic Conditions Using Microwave Techniques: A Review

**DOI:** 10.3390/s26020359

**Published:** 2026-01-06

**Authors:** Marek Ostrysz, Zenon Szczepaniak, Tadeusz Sondej

**Affiliations:** Faculty of Electronics, Military University of Technology, 00-908 Warsaw, Poland

**Keywords:** microwave techniques, non-contact monitoring, vital sign detection, radar biomedical sensing, bioradar technology, Doppler radar, ultra-wideband radar, millimeter-wave radar, microwave reflectometry

## Abstract

This article reviews recent advances in microwave and radar techniques for non-contact measurement of human vital signs in dynamic conditions. The focus is on solutions that work when the subject is moving or performing everyday activities, rather than lying motionless in clinical settings. This review covers innovative biodegradable and flexible antenna designs for wearable devices operating in multiple frequency bands and supporting efficient 5G/IoT connectivity. Particular attention is paid to ultra-wideband (UWB) radar, Doppler sensors, and microwave reflectometry combined with advanced signal-processing and deep learning algorithms for robust estimation of respiration, heart rate, and other cardiopulmonary parameters in the presence of body motion. Applications in telemedicine, home monitoring, sports, and search and rescue are discussed, including localization of people trapped under rubble by detecting their vital sign signatures at a distance. This paper also highlights key challenges such as inter-subject anatomical variability, motion artifacts, hardware miniaturization, and energy efficiency, which still limit widespread deployment. Finally, related developments in microwave imaging and early detection of pathological tissue changes are briefly outlined, highlighting the shared components and processing methods. In general, microwave techniques show strong potential for unobtrusive, continuous, and environmentally sustainable monitoring of human physiological activity, supporting future healthcare and safety systems.

## 1. Introduction

In recent years, there has been a dynamic development of technologies enabling contactless monitoring of human vital functions [1,2]. One of the most promising methods in this field is bioradar technology [3,4], which combines advanced radar techniques with biomedical signal analysis. Its undeniable advantage is the ability to perform measurements in a non-contact manner, even through physical obstacles, which opens new perspectives for applications in healthcare, rescue operations, and security systems [5]. The development of this technology is supported by advances in signal processing, device miniaturization, and artificial intelligence or smart integrated design [6]. As a result, it has become possible to track parameters such as respiration, pulse, and heart activity with increasing accuracy and reliability, even under challenging environmental conditions.

Bioradar technology is a modern, contactless method for monitoring vital parameters, capable of detecting subtle physiological movements such as breathing and heartbeat, even through non-metallic obstacles, making it useful in medicine, rescue operations, and public safety [7,8]. A precise biomedical radar [9] allows the measurement of vital parameters with micrometer accuracy, and by synchronously recording ECG (Electrocardiography) and radar signals in patients with cardiac arrhythmias, it enables innovative arrhythmia classification techniques with over 80% accuracy through the use of nonlinear dimensionality reduction and a neural network based on distributional encoding.

Contactless monitoring of vital signs using advanced radar techniques [10], such as the Extended Noise-Immune Motion Sensing (ENIMS) method based on a coherent low-IF CW radar, allows a significant increase in accuracy and noise immunity, allowing precise detection of breathing and heartbeat at distances up to 3.2 m in complex and crowded environments with very low transmission power. This has important medical applications, especially in the diagnosis of sleep apnea and heart diseases, and supports the development of radar technology for everyday and home applications.

The phased-MIMO technology integrated with a 77 GHz FMCW (Frequency-Modulated Continuous Wave) radar [11] enables steering of mmWave beams in different directions and simultaneous monitoring of multiple targets, increasing angular resolution and signal-to-noise ratio through the use of multiple transmit-receive antenna pairs, making it an effective solution for contactless tracking of vital signs of multiple people in crowded environments such as hospital rooms.

Microwave radiation can affect implanted pacemakers by generating heat and acoustic waves that cause microphone oscillations in the device, producing artifacts in ECG signals [12]. However, modern pacemakers are designed to resist typical electromagnetic fields, and their operation is not harmed by such exposure, although further research is needed on specific exposure conditions.

The rest of this paper is organized as follows. Section 2 introduces the theoretical foundations of microwave technologies and methods, including their interaction with biological tissues and their main biomedical and industrial applications. Section 3 analyzes the research status and publication trends based on a comprehensive PubMed literature review covering the years 1949–2025. Section 4 presents applications of microwave techniques for non-contact vital sign monitoring under dynamic conditions, with particular emphasis on radar-based solutions and advanced signal processing methods. Section 5 discusses the main challenges and limitations of contactless vital sign measurement using microwave and radar technologies. Section 6 outlines development perspectives, focusing on biodegradable and flexible antennas, wearable devices, telemedicine systems, and advanced microwave imaging approaches. Finally, Section 7 provides a summary of the reviewed technologies and indicates directions for future research.

## 2. Current Microwave Technology Applications

Microwaves are short-wavelength electromagnetic waves that are used in many fields such as telecommunications, radar, GPS navigation, and microwave heating [13,14,15]. They are characterized by their ability to transmit large amounts of data due to high frequency [16,17] and the capability to form narrow antenna beams [18,19]. They are a key element of modern point-to-point communication systems [20] and are widely used to transmit voice, images, and data over long distances.

The theoretical foundations and microwave methods are based on the use of short-wavelength electromagnetic waves whose propagation in a medium is governed by absorption, scattering, and the (generally complex) dielectric permittivity of the material. Microwave imaging [21,22] exploits the analysis of signals reflected from and transmitted through regions with different dielectric properties, enabling non-invasive detection of abnormalities such as tumors. This process is supported by advanced calibration procedures, interference-suppression techniques, and image-reconstruction algorithms that together allow the precise localization of pathological changes.

Moreover, microwaves have found wide applications in industry and scientific research [23], where they are used for rapid and efficient heating, material drying, enhancement of chemical reactions, as well as food processing and ceramic sintering. Microwave technology allows significant energy savings and reduced process time [24], which contributes to its growing popularity as an innovative tool in various industrial sectors. The use of microwaves combined with modern catalysts and precise process control opens new possibilities for eco-friendly and efficient production.

In addition to their extensive use in communications [25,26] and industry [27,28], microwave technology has also found an important place in modern defense systems [29,30], where high-power microwave (HPM) weapons use concentrated electromagnetic pulses to disrupt [31] and destroy enemy electronic systems, representing a breakthrough in military strategy. Furthermore, HPM weapons are characterized by high precision and immediate action, enabling effective neutralization of electronic systems and threats such as drones without causing the collateral damage typical of conventional weapons. Moreover, the ability of HPM systems to selectively target electronic components makes them particularly attractive for asymmetric warfare scenarios and the protection of critical infrastructure. As research in microwave sources and power generation continues to advance, the role of HPM technology in future defense architectures is expected to grow significantly, further reshaping modern military capabilities. At the same time, microwave technology underpins one of the most fundamental defense applications—military radar systems—which rely on microwave signals for the detection, tracking, and identification of airborne, maritime, and ground targets. These radar systems are essential for maintaining situational awareness, providing early warning, and ensuring effective protection against conventional and asymmetric threats.

Ferroelectric varactors [32,33], based on complex metal oxides such as barium titanate or potassium niobate [34], exhibit dielectric permittivity dependent on the DC field, which makes them attractive components for microwave technologies. Their properties allow for the creation of electronically tunable filters and antennas, which are crucial for agile communication and radar systems. The commercialization of ferroelectric varactors is developing rapidly, offering new design and system-level opportunities in modern microwave electronics.

Meanwhile, recurrent neural networks (RNNs) represent a modern tool used in microwave and electromagnetic fields for modeling nonlinear devices and solving problems related to signal scattering. The use of LSTM (Long Short-Term Memory) models enables effective prediction of electromagnetic field behavior in the time domain and precise representation of power amplifier characteristics with memory, significantly improving optimization and control processes in microwave devices. As a result, these techniques contribute to the increased efficiency and reliability of modern microwave systeWms.

Microwave photonic filters (MPFs) [35,36] are key components of modern photonic systems, offering advanced detection and measurement techniques widely used in aviation and other high-performance measurement systems. The study [37] presents an analysis of their classification, characteristics, and reconfiguration, as well as detection methods that enable precise measurements of the frequency and angle-of-arrival, emphasizing the growing importance of MPFs in modern aerospace systems.

Microwave filters are crucial components in wireless communication systems, enabling selective signal frequency selection and suppression of other interferences. Due to limited spectrum allocation, there is an increasing demand for reconfigurable microwave filters, which, thanks to tunability and multi-band operation capability, offer flexibility and high performance. The article [38] reviews the latest design techniques and applications of these filters. Photonic instantaneous microwave frequency measurement (IFM) uses a pair of microwave filters with complementary frequency responses, providing a wide measurement range and high accuracy. Experimentally, frequency measurement up to 36 GHz has been achieved [39] with accuracy better than ±0.2 GHz, which is significant for rapid RF signal identification in radar and communication systems.

## 3. Research Status—Analysis of Publication Trends

To assess the development of research on the use of microwave techniques to monitor vital signs, a literature analysis was conducted using the PubMed database. The search was expanded to include indexed titles, abstracts, and full-text publications using the query “vital signs AND microwave”. This review covered the years 1949–2025 and identified 641 publications related to this topic.

**Figure 1:**
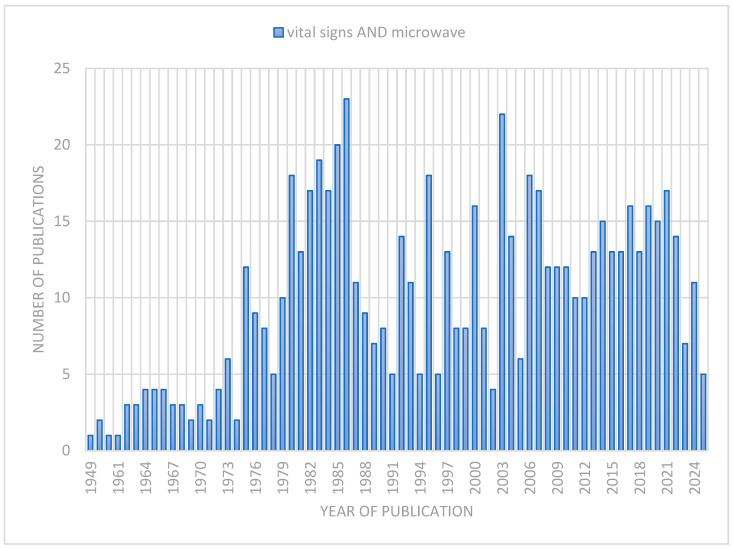
Number of publications on vital signs measurement using microwave techniques in PubMed database.

The analysis of the number of publications concerning the measurement of vital signs using microwave techniques in the PubMed database presented in Figure 1, shows a clear increase in interest in this topic since the 1980s, with notable peaks in 1986, 2003, and 2006–2007. The decrease in the number of publications in 2023 and 2025 may result from delayed indexing or incomplete data, especially for the year 2025.

Analyzing the number of publications, it was observed that in the 1940s and 1950s the topic of microwaves in the context of vital sign monitoring was very limited, with only a few publications per year (for example, in 1949—one publication). It was not until the 1970s, especially after 1975, that the number of publications began to gradually increase, reaching 18 in 1980 and 23 in 1986. This period marked the intensive development of microwave technologies that contributed to the growing number of studies in this field.

From the beginning of the 21st century, research maintained a high level of interest. However, beyond quantitative growth, a qualitative shift in research focus is evident. Although earlier studies primarily concentrated on hardware feasibility and antenna design, the recent literature demonstrates a dominant trend towards advanced signal processing. There is a notable surge in publications utilizing Machine Learning and Deep Learning techniques to address motion artifacts and separate multi-target signals, reflecting the maturation of the technology from experimental setups to attempts at robust applications.

Nevertheless, a critical analysis of these trends reveals a significant ‘translation gap’. While the volume of academic research has grown exponentially, the transition to commercially available medically certified devices remains disproportionately slow. This discrepancy indicates that while the theoretical principles (discussed in Section 2) are well-established, the field is currently facing a ‘robustness bottleneck’. The majority of studies are still conducted in controlled laboratory environments, and the sharp increase in recent publications reflects the community’s intense struggle to solve the practical challenges of random body movement and standardization (further analyzed in Section 4 and Section 5), rather than widespread clinical adoption.

Trend analysis indicates sustained and growing interest in microwave technologies for non-contact vital sign monitoring, which may result from the advancement of modern contactless systems, device miniaturization, and the integration of microwave technologies with artificial intelligence and deep learning algorithms.

Interest in this topic is also driven by the development of remote health monitoring and telemedicine systems, especially in the context of new health challenges and the growing need for remote care.

## 4. Applications in Dynamic Conditions

In the field of vital sign monitoring, microwave methods are gaining increasing importance, offering non-invasive and contactless solutions that surpass traditional techniques that require physical contact with the patient [40]. An example of an innovative approach is the “microwave stethoscope” (MiSt) presented by Celik et al. [41], which uses a single wideband microwave sensor operating in the 700 MHz to 1.5 GHz range. The method is based on measuring the microwave reflection coefficient from the chest, which, despite lower sensitivity compared to the transmission technique, is compensated by an optimized sensor design ensuring better skin coupling and advanced digital signal processing (DSP) algorithms. MiSt enables simultaneous monitoring of respiratory rate, pulse, and lung water content, which is clinically significant, particularly for detecting pulmonary edema and monitoring patients after cardiopulmonary surgery. The experimental results obtained from phantom tests, computer models, and clinical studies confirm the high accuracy and reliability of these measurements, as well as the ability to record signals similar to an electrocardiogram.

As highlighted in [42], two types of ultra-wideband microwave sensor technology are used for non-invasive monitoring of vital signs and lung water levels. The first consists of radiating sensors operating in the 1.5–10 GHz range, which can be integrated with textiles to improve comfort. The second consists of non-radiating coupled sensors operating in the 1.3–10 GHz range, characterized by higher signal fidelity and reduced susceptibility to external interference. Both technologies employ deep learning-based denoising, improving measurement quality, especially under patient motion. In practice, coupled sensors demonstrate superior accuracy and stability, making them a more effective solution for medical monitoring.

In [43], two types of wearable microwave antenna sensors for monitoring vital signs and lung water levels were compared: narrowband (NB) and ultra-wideband (UWB). The NB sensor has a bandwidth of 240 MHz and is fed by a microstrip offset-feed transmission line, while the UWB sensor operates in the 1.5–10 GHz range, using a CPW feeding line and a U-shaped radiator. Both sensors are fabricated on a flexible cotton textile substrate. Their safety was confirmed by specific absorption rate (SAR) measurements compliant with IEEE standards. Tests using simple detection algorithms showed that UWB sensors provide higher accuracy and better performance in detecting heart rate, respiration, and lung water levels compared to NB sensors.

Multipoint vital sign monitoring [44,45,46] is essential to obtain detailed information about physiological changes, while traditional single-sensor approaches [47,48] are insufficient to capture multipoint vibrations. Existing contact-based methods can cause discomfort and allergies, whereas contactless optical and acoustic methods are prone to interference. To address these challenges, the MultiVital system [49] was developed, utilizing mmWave MIMO (Multiple Input, Multiple Output) radar for synchronous, contactless, multipoint monitoring of vital signs. This system measures vibrations on the chest surface at multiple points, integrating seismocardiography (SCG) and electrocardiography (ECG) sensors for validation. Through advanced algorithms and signal processing, MultiVital enables the precise detection of subtle cardiopulmonary movements in different body areas, providing a more accurate and comprehensive assessment of cardiopulmonary health.

Article [50] describes the use of an impulse-radio ultra-wideband (IR-UWB) radar for precise, contactless real-time monitoring of heart rate and respiration rate. The study was conducted on 50 patients in a cardiac clinic who were monitored both by radar and by reference methods such as electrocardiography and capnography. The radar operated in the 6.5–8 GHz band and allowed measurements from a distance of 1.5 m, ensuring comfort and safety for users. The results showed high consistency in the respiration measurements and moderate accuracy in the heart rate, with better performance in the lying position. The study accounted for the impact of body posture and vital sign values on measurement precision. IR-UWB proved resistant to interference and body motion, making it a promising solution for long-term and safe health monitoring, especially in conditions that limit physical contact.

Modern vital sign monitoring methods use advanced radar technologies, including millimeter-wave (mmWave) systems [51,52], which offer high resolution and precision in detecting subtle movements such as breathing and heartbeat. Operating in the 30–300 GHz range, mmWave radars enable contactless, continuous real-time health monitoring, resistant to light interference and physical obstructions. This technology can be integrated into everyday devices, such as smart home systems, supporting early disease detection and monitoring of elderly or ill individuals. Advanced algorithms like DR-MUSIC, which employ adaptive filtering and spectral analysis, suppress respiratory motion artifacts and enhance heartbeat signals, significantly improving heart rate accuracy even under low signal-to-noise conditions. Experimental studies [53] confirm the high effectiveness of this method in various environments, including at distances up to 1.5 m, making mmWave radar a promising tool for precise vital sign monitoring in both medical and consumer applications.

The growing demand for health monitoring has driven the development of contactless vital sign monitoring technologies that offer comfort and high precision. In response, the MRVS method [54], based on millimeter-wave FMCW radar, was developed to detect chest movements while separating respiration and heartbeat signals. The process consists of three stages: signal processing, decomposition, and reconstruction. The mmWave radar first detects chest movements, and through signal overlapping and phase differentiation, removes static and harmonic breathing interference while enhancing heartbeat signals. Next, the discrete wavelet transform (DWT) reduces residual noise and decomposes the signal, followed by an adaptive Kalman filter (AKF) with root normalization to accurately reconstruct the signal, allowing precise heart rate estimation. Experiments using radars operating in the 76–81 GHz band confirmed the effectiveness of the MRVS method, achieving measurement errors below 7% across different angles, distances, and body positions. This approach represents a modern, non-contact, and highly precise solution for vital sign monitoring in medicine and healthcare systems.

To meet the challenge of monitoring multiple individuals simultaneously, the advanced VSDR (Vital Signs-based Dictionary Recovery) method [55] was developed. Using a sparsity-based signal model and specific cardiopulmonary features, it enables precise localization and contactless monitoring of respiration and heartbeat for multiple people at once. This method performs effectively even in complex and cluttered environments with multiple objects, using only a single-channel SISO FMCW radar. Through dictionary-based analysis of high-resolution grids corresponding to cardiopulmonary features, VSDR identifies breathing and heart rate frequencies, achieving higher precision than previous methods. Tests conducted on data from 30 subjects confirmed its effectiveness and superiority, making it a promising tool for efficient, contactless health monitoring in medical facilities and telemedicine systems.

Contactless vital sign monitoring technologies are becoming increasingly important, especially in terms of comfort and reducing physical contact with patients [56,57]. One such innovation is the use of ultra-wideband (UWB) radars for monitoring heart rate variability (HRV) and mental state [58]. Studies [59] have confirmed that time-domain parameter measurements using the UWB radar are reliable, although frequency-domain parameters are more prone to interference at low signal-to-noise ratios. Importantly, heart rate variability analysis also allowed an accurate differentiation of mental states with more than 82% accuracy, opening new possibilities for non-contact, non-invasive mental health monitoring. These achievements indicate promising applications of UWB radars in medicine and healthcare.

An important complement to modern vital sign monitoring technologies is the self-calibrating Doppler radar system [60], which measures heart rate and respiration in a contactless manner while ensuring patient comfort. The system is based on a four-layer hardware-software architecture, where baseband signal modeling enables accurate demodulation of chest movements. The advanced signal identification method was formulated as a constrained quadratic minimization problem solved using linear matrix inequality (LMI) relaxation techniques. As a result, the system automatically adapts to measurement conditions, ensuring reliable vital sign monitoring confirmed by numerous experimental tests.

Recent advances [61] in electromagnetic sensor technologies open up broad possibilities for health monitoring, diagnostics, and continuous patient observation. These technologies enable the detection of changes in tissue dielectric properties, allowing non-invasive imaging and monitoring of vital functions. The use of advanced materials, antennas, and signal processing in electromagnetic sensors paves the way for the development of modern, safe, and precise medical tools. However, despite numerous advantages, clinical implementation still faces challenges due to diverse conditions and complex signal characteristics which require further research and optimization.

Electromagnetic-acoustic (EMA) detection techniques [62] combine the benefits of radar and ultrasound, enabling complete, non-contact monitoring of physiological and bioelectrical signals in real time. EMA is particularly suitable for long-term health supervision in home and remote environments, supported by advanced algorithms and artificial intelligence, opening new opportunities in healthcare. Three main types of EMA sensors are distinguished: chip-based radar sensors, thermoacoustic measurement devices, and photoacoustic detection and imaging systems, all employing dedicated algorithms for signal analysis in different domains. This synergistic integration of electromagnetic and acoustic technologies represents a promising tool for modern medicine and related applications.

In the context of cardiovascular diseases, congestive heart failure (CHF) remains one of the leading causes of death worldwide [63,64], highlighting the importance of effective detection and monitoring of this condition. In response, a portable microwave imaging system [65] was developed that combines a compact antenna array with high-speed data acquisition to create microwave heart images. This system offers a non-contact, safe, and low-cost method that surpasses traditional technologies such as X-ray and MRI, and its portable design allows rapid deployment in various environments, including outside hospital settings. The presented studies confirm the growing effectiveness and maturity of microwave technologies for contactless vital sign monitoring, particularly under dynamic conditions. The use of advanced signal processing algorithms and integration with radar and artificial intelligence technologies [66] enables precise multidimensional measurements, opening new perspectives for preventive medicine, home healthcare, and telemedicine [67].

A particularly critical dynamic scenario is the real-time assessment of driver status, where the human factor is a primary cause of accidents [68]. In this environment, contactless monitoring faces dual interference from the driver’s voluntary movements and vehicle vibrations. To address signal contamination where traditional methods such as FFT fail, recent CW Doppler studies [69] propose the Multiple Signal Classification (MUSIC) algorithm, which experimentally outperforms FFT in accurate heart rate estimation even with heavy motion artifacts.

Furthermore, the robustness of monitoring systems can be enhanced through optimized hardware configuration and advanced processing frameworks. Research employing FMCW radar [70] highlights that optimal sensor placement—determined by Signal-to-Clutter Ratio (SCR) analysis—is fundamental for signal quality. When combined with phase processing and Deep Learning models, such systems have achieved HR and HRV estimation accuracy exceeding 90% compared to reference ECGs, even in mild motion scenarios.

Moving towards integrated solutions, a novel two-stage method using a 60 GHz FMCW radar mounted on the sun visor has been developed [71]. This approach performs a coarse estimation using Discrete Wavelet Transform (DWT) followed by a refined estimation using a Relevance Vector Machine (RVM), stabilized by a Sequential Kalman Filter (SKF). This architecture allows for reliable HR measurements in very short windows (5 s) under varying conditions, validating the effective transfer of biological perception principles to automotive sensing.

## 5. Challenges and Limitations of Microwave Vital Sign Monitoring

Basic vital signs such as heart rate, blood pressure, temperature, and oxygen saturation are key indicators for assessing a patient’s health status, but their non-invasive measurement in dynamic and varied conditions still faces many challenges and limitations [72]. Non-invasive glucose concentration measurements from the fingertip using radio and microwave frequencies [73] are promising due to the high blood availability and relative homogeneity of the biological layers. However, their precision is limited by small changes in the blood’s electrical permittivity caused by glucose levels, as well as individual variations in skin thickness and fingerprint patterns, which can lead to significant discrepancies in the measurements. In practice, there is a high risk of error, and currently available non-invasive devices are not considered by experts [74,75] to be accurate enough for therapeutic decision-making, so patients should rely on certified invasive methods such as traditional glucometers.

Continuous and rapid heart rate (HR) detection is crucial for assessing heart rate variability (HRV), which reflects the activity of the autonomic nervous system and changes in sleep stages [76,77]. However, precise HR measurement under dynamic conditions is challenging due to frequency resolution limitations and algorithmic complexity [78]. In [79], an innovative method based on the Fourier-Bessel Series Expansion (FBSE) with respiratory noise reduction was proposed, allowing fast and accurate heart rate detection in less than 5 s without the need for filtering. Experiments using a 24 GHz Doppler radar demonstrated good agreement between HRV results obtained with this method and those from polysomnography, suggesting great potential for automatic sleep stage classification and daily vital sign monitoring.

Doppler cardiograms (DCGs) remotely detected using Doppler radar (DRS) provide detailed information about heart motion, but traditional methods require breath-holding to avoid interference. A high-linearity continuous-wave (CW) radar with digital tuning and a parameterized respiration filter (PRF) algorithm was developed [80], effectively removing respiratory artifacts and allowing precise detection of DCG. Simulations and experiments confirmed that PRF outperforms conventional methods in signal extraction. Granular differential DCG (D-DCG) shows great potential in HRV analysis and cardiac disease diagnosis, making this technique a convenient and reliable tool for both daily and clinical heart health evaluation. Most heart activity measurement devices, such as electrocardiograms (ECG), rely on contact electrodes, that cause discomfort and limit their use. Based on magnetic resonance analysis, it was determined [81] that a single radar sensor can remotely detect a Doppler cardiogram (DCG) from a distance of up to 1 m, capturing Doppler signals generated by cardiac motion from the back of the chest. This DCG provides full temporal information corresponding to the P, QRS, and T waves of the ECG. Thus, a miniature remote sensor can serve as a portable device for patients (e.g., burn victims), expanding applications in personal care, rescue, and other domains.

Heart rate variability (HRV) is an important indicator of stress and general health, but standard ECG-based methods are cumbersome for long-term monitoring due to the need for contact electrodes [82,83]. To overcome these limitations, microwave reflectometric techniques [84] have been developed, which allow contactless and non-invasive measurement of vital parameters, including HRV, based on analysis of electromagnetic wave reflections from the chest. The signal from a microwave reflectometer [85], processed through a quadrature phase detector, allows extraction of both phase and amplitude components, which are then analyzed using the fast Fourier transform and the wavelet transform. This approach enables the evaluation of heartbeat and respiration frequency spectra, opening new possibilities for daily health monitoring and stress assessment. Despite promising results, this technology still requires further development in advanced signal processing and consideration of individual anatomical differences.

In addition, Doppler motion detection systems [86] offer further possibilities for contactless monitoring of vital signs such as respiration and heartbeat. These solutions consist of a Doppler radar transceiver, an A/D converter, and dedicated digital signal processing (DSP) software that enables the precise extraction of the body motion velocity without the need for sensors attached to the patient. Using compact, affordable, and commercially available motion sensors, this system provides an economical and practical solution for both clinical and everyday health monitoring applications.

Furthermore, microwave systems [87] for vital sign detection are being developed, which operate by illuminating the observed object with low-intensity microwave beams and analyzing backscattered signals from the body. Such systems, operating in the X-band, can detect heartbeat and respiration at distances of about 30 m, even through obstacles such as cinder block walls. These solutions are characterized by high sensitivity and suitability for rescue and contactless diagnostic scenarios. Experimental results confirm their potential for life detection in hard-to-reach or critical situations. Moreover, microwave techniques are also used to record apexcardiograms. This method [88,89] involves detecting changes in reflected microwaves caused by the motion of the chest wall associated with left ventricular activity. Studies indicate that this technique enables the precise identification of subtle precordial motion structures, which can significantly support advances in cardiac diagnostics.

The most advanced approaches use deep learning techniques to reconstruct electrocardiogram (ECG) signals from radar data. In recent studies [90], a signal model incorporating subtle cardiac activity features detected by radar was proposed, together with the radarODE architecture that combines classical differential equations with a deep neural network to extract temporal and morphological features. This approach allows ECG signal reconstruction with high accuracy, even during body movement, greatly increasing the potential of radar-based heart monitoring in real clinical and home environments.

Among the emerging directions in radar microwave technologies, biometric authentication systems [91,92] play an important role, utilizing users’ physiological characteristics such as unique cardiac patterns [93,94]. An example of such a solution is the HeartPrint system [95], which enables continuous contactless identification of multiple users in environments such as smart homes or offices using a single millimeter-wave radar. This system records micro-movements of the skin surface caused by heartbeat, using them as a biometric feature. Through user position clustering and signal feature extraction, it is possible to separate and identify individual cardiac signals even in the presence of multiple people. The achieved authentication accuracy exceeds 95%, and the spoofing attack success rate is below 3%, making this solution a promising alternative to traditional biometric methods in the context of security and privacy.

To provide a concise overview of the main microwave techniques discussed in this section, Table 1 summarizes their typical configurations, measured vital parameters, key advantages, and major limitations in the context of non-contact vital sign monitoring.

Detecting vital signs in dynamic conditions requires a fundamental shift in system design principles compared to static monitoring. While static measurements primarily prioritize sensitivity to micro-vibrations (sub-millimeter displacements), dynamic scenarios demand a system architecture capable of robustly handling macroscopic movements ranging from centimeters to meters. From a hardware perspective, this necessitates the implementation of High Dynamic Range (HDR) receivers to prevent saturation caused by strong, fluctuating reflections from moving limbs or environmental clutter. Furthermore, unlike static setups that benefit from high-gain narrow beams, dynamic systems often require wider beamwidths or Adaptive MIMO beamforming to spatially track the subject and maintain signal integrity. Carrier frequency selection also involves a critical trade-off, while millimeter-wave frequencies (e.g., 60–80 GHz) offer superior sensitivity to chest displacement, they are significantly more susceptible to phase wrapping errors during large body movements compared to lower microwave bands (e.g., 2.4–10 GHz). Consequently, in terms of signal processing, simple spectral analysis methods such as FFT become insufficient due to the smearing of the Doppler spectrum. Robust detection in non-stationary environments therefore requires a multi-stage approach, combining motion artifact cancelation via adaptive filters (e.g., LMS, RLS) or Blind Source Separation (BSS) with advanced tracking algorithms, such as Kalman filters, to distinguish quasi-periodic vital signs from aperiodic motion noise. A systematic comparison of these critical hardware and processing requirements is presented in Table 2.

## 6. Development Perspectives

The development of IoT sensor technologies using biodegradable and renewable materials in microwave antennas [96,97] represents a key step toward sustainable growth, reducing e-waste and expanding sensor applications in fields such as agriculture, environmental monitoring, healthcare, wearable electronics, logistics, and food processing, while also addressing design, manufacturing, and future development challenges. The rapid progress of 5G technology and the growing demand for wearable devices drive the advancement of high-performance flexible microstrip antennas, such as the design proposed in [98], which uses denim and cotton substrates combined with copper as conductive material. Operating below 6 GHz, this antenna ensures optimal communication with low power consumption. Simulations in CST Studio Suite confirmed its high mechanical flexibility, strong radiation characteristics, and excellent operational parameters, making it ideal for modern wearable 5G applications.

Modern microwave imaging systems are gaining popularity as safe, non-ionizing, and non-invasive alternatives to traditional diagnostic methods [99], using differences in the dielectric properties of healthy and cancerous tissues [100] for effective tumor detection. The microwave imaging system developed in [101], using a printed UWB dipole antenna at 5.8 GHz, enables affordable, portable, and safe image reconstruction for tumor detection, confirmed experimentally in phantoms mimicking healthy and malignant tissues. Modern tumor imaging techniques [102], such as MRI, PET, and CT, offer high resolution and diagnostic precision, but their cost and limited accessibility, especially in developing countries, highlight the need for more affordable, portable, safe, and non-invasive technologies such as microwave imaging systems using UWB antennas for effective cancer detection.

The rapid development of telemedicine systems and services [103,104] worldwide significantly improves access to professional healthcare, improves quality, and supports preventive medicine through early intervention, while also reducing healthcare costs [105], as confirmed by pilot programs and justifying further investment in this technology [106]. In [107], an innovative wideband foldable antenna was presented, designed specifically for the early detection of heart failure symptoms in the UHF band. With a compact 3D structure of 0.1λ × 0.29λ × 0.09λ, it achieves a peak gain of 4.2 dBi, a front-to-back ratio of 7–13 dB, and an efficiency exceeding 87% in the 560–1060 MHz range. Used together with a compact microwave transceiver, it forms an effective monostatic radar system whose heart failure detection efficiency was confirmed through tests on human body phantoms.

Article [108] discusses the latest advances in microwave technology applied to the diagnosis of congestive heart failure (CHF), emphasizing the importance of early and precise detection for effective treatment and the potential of non-invasive cardiac imaging. It analyzes various CHF detection techniques, focusing on their resolution and specificity, and indicate directions for future research to improve microwave-based cardiac diagnostics. A smartwatch antenna [109] operating in the 60 GHz band allows Wi-Fi signal reception without requiring a Bluetooth connection to a phone. Built on a flexible leather substrate, it is comfortable to wear and may support diagnostics of various health conditions. The tests of different antenna versions, considering tissue and bending effects, confirmed good performance and high potential for further development in medicine and telecommunications.

The study in [110] highlights that contactless vital sign detection technology using FMCW radar and the improved Ensemble Empirical Mode Decomposition (EEMD) method enables comfortable and precise monitoring of respiration and heartbeat. The use of a symmetric alpha distribution instead of a Gaussian one, along with static noise filtering, allows effective separation of life signals from environmental noise, confirmed experimentally at distances of 0.5–2.5 m. This technology operates independently of lighting and environmental conditions, making it a versatile solution for vital sign monitoring in various applications. One such application [111,112,113] involves locating living persons trapped under rubble or behind obstacles, where Doppler radar enables completely non-contact detection of respiration and heartbeat. The growing importance of this technique [114] results from the rapid advancement of hardware and signal processing methods, which improve detection accuracy while improving the energy efficiency of rescue systems crucial in emergency situations. Alternatively [115], a single-channel 24 GHz CW radar using the maximal overlap discrete wavelet transform (MODWT) offers a simpler and cheaper architecture than multichannel FMCW or UWB systems, enabling effective detection of one or two people’s heartbeats through various barriers with accuracy greater than 95%. Although it does not measure range or direction, this method is particularly useful in applications such as search-and-rescue operations, medical monitoring, or security surveillance.

The use of GSM RF components in non-communication applications such as medicine [116,117] and science can offer a favorable cost-to-performance ratio. For instance [118], a dual 915 MHz/60 W microwave generator prototype costs under 250 USD and performs well in both thermal and non-thermal applications. Using legacy RF components can be advantageous, as it simplifies design, shortens production time, reduces cost, and still delivers high performance. More than 55 years ago, Herbert Fröhlich proposed [119] that very weak microwave signals could affect biological processes and that their coherent oscillations might play a significant role in detecting abnormal cells long before the appearance of cancer symptoms. If such “electromagnetic triggers” are identified, it may become possible to develop technologies for the early detection of cellular changes, potentially revolutionizing cancer diagnostics and therapy.

## 7. Summary

This article presents a comprehensive review of the latest achievements and challenges in non-contact vital sign monitoring using microwave and radar technologies. It describes innovative solutions in the field of biodegradable and flexible microwave antennas, which are key components in the development of modern wearable devices and the Internet of Things (IoT). Renewable and eco-friendly materials used in antenna design not only help reduce e-waste but also open new possibilities in areas such as agriculture, environmental monitoring, healthcare, and logistics. Moreover, the rapid advancement of 5G technology and the growing demand for high-performance, flexible microstrip antennas pose new challenges to designers, which have been addressed through the use of innovative materials such as denim and cotton substrates.

Particular attention is given to microwave medical imaging systems, which offer a safe, non-ionizing, and cost-effective alternative to traditional diagnostic methods such as MRI, PET, and CT. The technologies described, based on UWB antennas and radar systems, provide promising capabilities to detect cancerous changes and diagnose heart diseases while reducing costs and improving accessibility, especially in regions with limited medical resources.

This article also discusses advanced methods for monitoring vital signs such as heart rate, respiration, and heart rate variability (HRV), which achieve greater precision through the application of deep learning techniques, advanced signal processing, and Doppler radar systems. Their importance for telemedicine and everyday health monitoring is emphasized, particularly in dynamic conditions where traditional invasive methods are uncomfortable or impractical. These technologies also enable life detection and monitoring in crisis situations, such as the location of people trapped in rubble, representing a major advancement in rescue operations and public safety.

Despite numerous advantages and rapid development, this article highlights the existing limitations of these technologies, including the influence of individual anatomical differences, environmental interference, and the complexity of signal analysis algorithms. This underscores the need for further research and development, particularly in advanced data processing and measurement personalization, to improve the accuracy and reliability of such systems.

From a future perspective, the authors emphasize the importance of integrating microwave technologies with ecological solutions and their potential in early detection of cellular changes, which could revolutionize methods for identifying cancer and other disorders. Examples of innovative biometric systems based on millimeter-wave radar also demonstrate new possibilities in security, privacy, and contactless user identification, with wide-ranging applications in smart living and working environments.

A key advantage of microwave sensing is its non-invasive and contactless nature: measurements can be performed without direct skin contact and without the need for adhesive electrodes or coupling gels. By appropriately selecting the operating frequency, the penetration depth of the electromagnetic wave can be tailored to the target tissue or organ, enabling flexible optimization of measurement conditions. Furthermore, microwave systems are capable of monitoring vital signs through clothing, which is particularly relevant for field medicine, medical triage, and mass-casualty incidents, where rapid, contactless evaluation of a large number of patients is required.

In conclusion, the development of microwave and radar technologies for non-contact health monitoring represents one of the most promising and rapidly advancing fields in modern biomedical engineering and consumer electronics. These technologies not only contribute to improving quality of life and improving safety but also support sustainable development by offering more affordable, accessible, and eco-friendly diagnostic and monitoring solutions. The solutions presented in this article form the foundation for future innovations and indicate research directions that can significantly impact medicine, healthcare, and broader environmental and social monitoring.

## Figures and Tables

**Table 1:** Comparison of microwave techniques used for non-contact vital sign monitoring.

Microwave Technique	Typical Frequency/Configuration	Main Measured Parameters	Main Advantages	Main Limitations and Challenges
CW Doppler radar (including DCG and HRV)	Narrowband CW radar, typically 10–24 GHz, single or few antennas	Heart rate, Doppler cardiogram, respiration, HRV	Simple and low-cost hardware; high sensitivity to sub-millimeter chest motion; real-time operation	Susceptible to body motion and clutter; limited range and angular resolution; usually single-subject; requires careful calibration and phase unwrapping
FMCW/mmWave radar (incl. MIMO systems)	FMCW radar in the 60–81 GHz band, often MIMO arrays	Range-resolved heart and respiration rate, multi-person vital signs	Provides distance and angle information; supports simultaneous monitoring of several people; high spatial resolution	Higher hardware complexity and cost; demanding DSP and calibration; performance affected by clothing, orientation, and multipath
IR-UWB radar	Impulse-radio UWB, e.g., 6.5–8 GHz wideband pulses	Respiration rate, heart rate, HRV	High temporal resolution; good robustness to multipath; safe low-power operation	Large occupied bandwidth and regulatory constraints; moderate HR accuracy compared to ECG; requires sophisticated signal processing and denoising
Microwave reflectometry	Wideband reflectometer with quadrature phase detection	Heart rate, respiration, HRV, stress indicators	Fully contactless; extracts both amplitude and phase; relatively simple antenna configuration; suitable for long-term monitoring	Limited to short ranges near the chest; sensitive to posture and anatomical variability; needs advanced algorithms to separate overlapping cardiac and respiratory components
Microwave imaging systems (cardiac/pulmonary)	Arrays of UWB antennas around thorax, tomography, or radar imaging	Distribution of tissue permittivity, fluid accumulation (e.g., CHF, lung water)	Non-ionizing alternative to X-ray/CT; can visualize structural and functional changes; potential for early detection of disease	Complex multi-antenna hardware; computationally intensive reconstruction; sensitivity to modeling errors and patient motion; clinical validation still limited
Long-range life-detection X-band radar	CW or pulsed radar, 8–10 GHz, often with high-gain antennas	Presence of life, respiration, and coarse heart motion at tens of meters	Operation through debris and non-metallic walls; large detection range; useful in search-and-rescue scenarios	Limited physiological detail; prone to false alarms from moving clutter; bulky hardware and power requirements; not suited to everyday personal monitoring
Electromagnetic–acoustic (EMA) sensing	Hybrid EM and acoustic/thermoacoustic sensors, chip-scale radars	Combined mechanical, thermal, and bioelectrical activity	Simultaneous access to complementary modalities (radar + ultrasound/thermoacoustics); high information content; promising for home and telemedicine monitoring	Technology is still in the research stage; complex sensor design and signal fusion; calibration and safety aspects need further study before large-scale clinical deployment
Wearable/textile microwave sensors	Integrated narrowband or UWB antennas on flexible substrates (cotton, denim, textiles)	Heart rate, respiration, lung water level, sometimes ECG-like signals	Comfortable, unobtrusive, suitable for continuous monitoring; can be integrated into clothing; low power; compatible with IoT wearables	Performance depends on textile properties, body fit, and movement; SAR and safety constraints; fabrication variability; long-term durability and washing resistance must be addressed

**Table 2:** Comparison of hardware and processing requirements for static vs. dynamic vital sign monitoring.

Design Aspect	Static/Sitting Scenarios	Dynamic/Mobile Scenarios
Primary Challenge	Low Signal-to-Noise Ratio (SNR); detecting weak micro-vibrations.	Low Signal-to-Interference Ratio (SIR); suppressing strong motion artifacts.
Receiver Dynamic Range	Standard; clutter is mostly static and can be filtered.	High Dynamic Range (HDR); required to prevent saturation from fluctuating clutter.
Antenna Configuration	High-gain, narrow-beam antennas (fixed focus).	Wide-beam or Adaptive MIMO Beamforming (spatial tracking).
Preferred Frequency	mmWave (e.g., 60–80 GHz) for maximum sensitivity to displacement.	Lower Microwave (e.g., 2.4–10 GHz) to minimize phase wrapping errors during large movements.
Signal Processing	Basic Spectral Analysis (FFT, Peak Detection).	Multi-stage: Blind Source Separation (BSS), Adaptive Filtering (LMS/RLS), Kalman Tracking.
Motion Compensation	Not required (subject is still).	Critical; often requires Sensor Fusion (Radar + IMU/Camera).

## Data Availability

No new data were created or analyzed in this study. Data sharing is not applicable.
